# Trait coping styles and the maternal neural and behavioral sensitivity to an infant

**DOI:** 10.1038/s41598-022-18339-w

**Published:** 2022-08-23

**Authors:** Pilyoung Kim, Leah A. Grande, Alexander J. Dufford, Andrew Erhart, Rebekah Tribble, Tom Yeh

**Affiliations:** 1 Department of Psychology, University of Denver, Denver, 2155 South Race Street, Denver, CO 80208-3500 USA; 2 Department of Medical Social Sciences, Feinberg School of Medicine, Northwestern University, 633 N. St. Clair, Suite 1900, Chicago, IL 60611 USA; 3 Department of Computer Science, University of Colorado, Boulder, 1111 Engineering Drive, Boulder, CO 80309-0430 USA

**Keywords:** Emotion, Social behaviour, Stress and resilience, Human behaviour

## Abstract

During the postpartum period, new mothers experience drastic changes in their body, brain, and life circumstances. Stress from the emotional and physical demands of caring for an infant is associated with negative mood and parenting outcomes. The use of active coping strategies can increase mothers’ resilience during the postpartum period. However, little is known about the association between coping styles and maternal brain responses to infant cues. In the current study, we examined the associations among trait coping style, maternal brain responses, and behavioral sensitivity in a socioeconomically diverse sample of first-time mothers (N = 59). The use of more active trait coping strategies compared to passive coping strategies was associated with increased brain responses to infant cry sounds in brain regions that are critically involved in motivation and emotion regulation—substantia nigra, anterior cingulate gyrus, and inferior frontal gyrus. Increased brain activations in the midbrain and anterior cingulate gyrus were further associated with higher levels of maternal sensitivity observed during interactions with the infant. Thus, the findings provide support for mothers’ use of more active coping styles to promote neural and behavioral resilience for a positive transition to parenthood.

## Introduction

The arrival of a baby comes with joy as well as new responsibilities and demands. New mothers experience stressful events that are directly associated with their newborns, such as difficulties with birth and breastfeeding^1,2^. Mothers also experience stressful life changes such as reduced time for self-care, career or academic pursuits, and leisure time with their partner and friends^3,4^. In addition to caring for their infant, some mothers experience stressors such as financial difficulties, unsafe environments, violence, isolation, and limited social support^5,6^. Unfortunately, stress has been negatively associated with a mother’s ability to provide sensitive care for her infant^7,8^.

However, not all mothers are equally influenced by the demands of the early postpartum period, with some mothers exhibiting robust resilience^9,10^. In order to support those who are vulnerable, it is critical to understand what makes mothers more or less susceptible to stress and what makes them more or less resilient during the postpartum period. One resilience factor is coping style^11,12^. During and beyond the early postpartum period, coping strategies that are effective in managing negative emotions and stress have been associated with indicators of mothers’ well-being, such as lower levels of depressive or anxiety symptoms and more sensitive parenting^13,14^. However, the mechanisms by which these coping strategies enhance mothers’ resilience and functioning have not been well understood.

Individuals cope with stress in many different ways and often use multiple strategies depending on the situation^15^. Certain coping strategies have been associated with better outcomes and are thus considered more adaptive and effective. These strategies are characterized by active engagement with stressors and include cognitive reappraisal, reframing (e.g. giving a negative experience more positive meaning), seeking social support, and planning for solutions^16^. On the other hand, strategies that are passive and involve disengaging from stressors have been associated with more negative outcomes and are therefore considered maladaptive and ineffective^15,17^. These strategies include denying or avoiding the stressor and distracting oneself with unrelated activities. These strategies may help to reduce distress in the short term, but because they do not involve an effort to directly resolve the stressful situation, they tend to be less effective at improving negative emotions in the long term.

Similar to the general population, active coping in new mothers is associated with positive outcomes while passive coping is associated with negative outcomes^18^. Active coping has been prospectively associated with lower depressive symptoms and more sensitive parenting during the first postpartum year^13,14^. On the other hand, there is consistent evidence suggesting that passive coping is prospectively associated with higher depressive symptoms^1^ and less effective parenting^19^. Active coping such as cognitive reappraisal was associated with less negative emotional expressions during challenges^20^ while passive coping such as denial was associated with increased levels of stress hormones^21^.

Caring for an infant is a demanding task that requires the ability to manage negative emotional responses^22–25^. The brain and body of a new mother adapt in order to reduce reactivity to stress during the postpartum period. New mothers exhibit reduced physiological stress reactivity to acute stressors^26,27^. During the first month of the postpartum period, negative mood tends to decrease and positive perception about infants increases^28^. This psychological adaptation to motherhood is supported by brain plasticity. With the support of elevated oxytocin levels, mothers exhibit enhanced brain activation in response to their infants’ auditory cues such as cry sounds and visual cues such as facial expressions^29–32^. A wide network of brain regions is involved in response to infant cues, including the network that supports maternal motivation and reward responses composed of the nucleus accumbens, ventral tegmental area, as well as the substantia nigra, striatum, and medial prefrontal cortex (PFC). This brain network closely communicates with the salience network which includes the amygdala, anterior insula, and anterior cingulate cortex. The brain regions that are involved in emotion regulation, such as lateral PFC, and emotional and cognitive empathy, such as inferior frontal gyrus (IFG), insula, and superior temporal lobe, are also important to support sensitive parental responses to infants^33–35^. These brain regions are critical for activating mothers’ caring responses to infants^36–40^. On the other hand, mothers exposed to stress in the past or present show reduced brain activation in these regions when responding to their infants^7^. However, little is known about the association between coping strategies and brain responses to infant cues.

While the literature is limited, previous studies suggest that trait-like coping strategies and stress resilience are associated with enhanced brain responses in regions involved in motivation and emotion regulation^41–45^. The PFC plays a critical role in stress and emotion regulation. The PFC regulates the amygdala, a key region that detects threats and activates physiological and emotional stress responses^46^. More specifically, the ventrolateral PFC, dorsolateral PFC, medial PFC, and anterior cingulate cortex (ACC) implement explicit cognitive strategies such as cognitive reappraisal and reframing^47,48^. The PFC dysregulation during emotion regulation has also been observed in populations with mood dysregulation, including depression^49^ and anxiety disorders^50^. Active coping styles involve cognitive and behavioral controls that aim to downregulate negative emotional responses to stressors^45^. Thus, active trait coping styles were associated with the increased activation in ventromedial PFC in response to aversive images (e.g. violence, fear)^45^. Coping styles that involve active control in response to stressors have also been associated with increased structural volume in the ACC as well as lower anxiety and depressive symptoms, particularly among women^44^. Furthermore, active coping styles are related to more approach behaviors to stressors and effort to increase positive emotions. An active coping style such as cognitive reappraisal in response to negative images was also associated with increased activation in the nucleus accumbens, a part of reward and motivation neural circuitry^51^. During emotion regulation, women also exhibited greater engagement in the ventral striatum, which is also a part of the motivation and reward circuitry^52^.

Thus, in the current study, we examined the associations between trait coping styles and mothers’ brain responses to infant cues—particularly infant cry sounds. Cry sounds are effective at eliciting parenting-related brain activation, but they can also be perceived as negative and require emotional regulation from the parent. First, we hypothesized that a greater proportion of active versus passive coping would be associated with better parenting-related outcomes including lower parenting stress, lower negative mood, and higher maternal sensitivity. Second, we hypothesized that a greater proportion of active vs. passive coping would be associated with greater brain responses to infant cry sounds in regions that are important for motivation and emotion regulation including the nucleus accumbens, the ventral striatum, ACC, medial and lateral PFC. Third, we hypothesized that greater brain responses to infant cry sounds would further be associated with higher maternal sensitivity.

## Results

### Trait coping styles, maternal mood, and adaptation to motherhood

**Figure 1:**
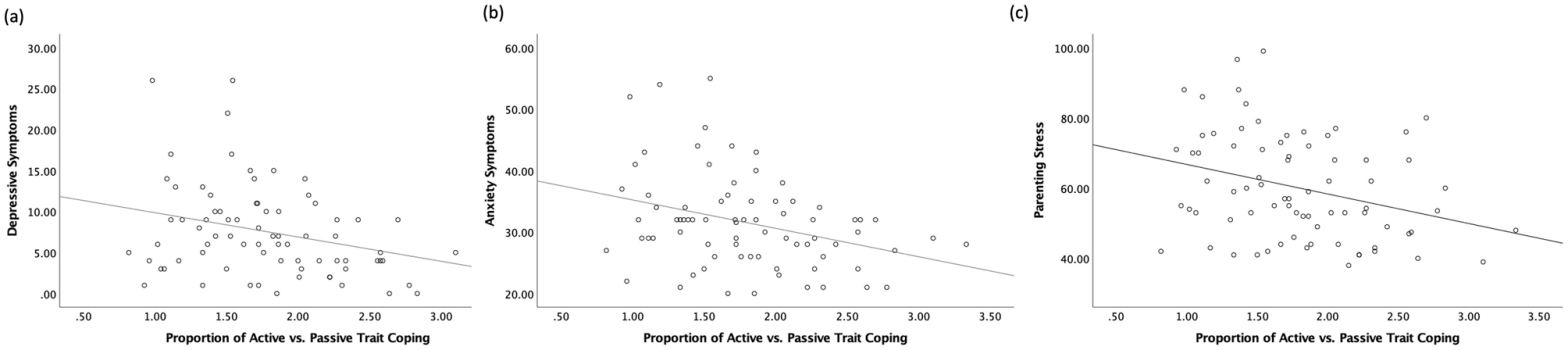
Scatterplots describing the associations between the proportion of active/passive trait coping styles and maternal postpartum outcomes in all participants (N = 77). Maternal postpartum outcomes included (**a**) depressive symptoms, *r*(75) = − 0.28, *p* = 0.01, (**b**) state anxiety symptoms, *r*(75) = − 0.32, *p* = 0.005, and (**c**) parenting stress, *r*(75) = − 0.32, *p* = 0.005.

The zero-order correlations among the variables are also provided in Tables S1 and S2. In all participants, the proportion of active/passive coping styles was negatively associated with both depressive symptoms, *r*(75) = − 0.28, *p* = 0.01, *q* < 0.05, and with state anxiety symptoms, *r*(75) = − 0.32, *p* = 0.005, *q* < 0.05 (Table S1; Fig. 1). The proportion of active/passive coping styles was also negatively associated with parenting stress, *r*(75) = − 0.32, *p* = 0.005, *q* < 0.05 (Table S1). The proportion of active/passive coping styles was not associated with maternal sensitivity. The only demographic variable that was associated with coping style was the use of psychotropic medication, *r*(75) = − 0.26, *p* = 0.03 (Table S1), but it did not survive a multiple comparison correction (*q* > 0.05).

In the subset of the participants with fMRI data, the proportion of active/passive coping styles was negatively associated with state anxiety symptoms, *r*(57) = − 0.26, *p* = 0.04 (Table S2), but it did not survive a multiple comparison correction (*q* > 0.05). The proportion of active/passive coping styles was not associated with parenting stress (p = 0.12) and maternal sensitivity (*p* = 0.46). No demographic variable (Table 1) was associated with the coping style variable (*ps* > 0.05). Analysis of differences between the sample included in the fMRI analysis (N = 59) and the sample not included in the fMRI analysis (N = 18) was included in the supplementary materials.

**Table 1:** Characteristics of the participants.

	Whole sample (N = 77)			fMRI sample (N = 59)		
	N(%)	Mean ± SD	Range	N(%)	Mean ± SD	Range
Maternal age (years)	–	25.96 ± 5.26	18–37	–	25.82 ± 5.45	18–37
**Maternal Race/Ethnicity**						
Caucasian	31 (40.3)	–	–	23 (39.0)	–	–
Hispanic	32 (41.6)	–	–	27 (45.8)	–	–
African-American	6 (7.8)	–	–	4 (6.8)	–	–
American Indian/Alaska Native	1 (1.3)			0 (0.0)		
Asian	2 (2.6)	–	–	1 (1.7)		
Other	5 (6.5)	–	–	4 (6.8)	–	–
Maternal education (years)	–	14.03 ± 2.49	7–20	–	14.08 ± 2.34	9–20
Infant sex (female)	40 (51.9)	–	–	35 (59.3)	–	–
Postpartum month at the time of home visits	–	3.58 ± 1.68	0.46–7.68	–	3.48 ± 1.68	0.46–6.96
Postpartum month at the time of fMRI scans	–	–	–	–	4.55 ± 1.92	0.89–10.66
Proportion of active/passive trait coping styles	–	1.80 ± 0.55	0.82–3.33	–	1.76 ± 0.54	0.82–3.10
Maternal sensitivity	–	5.25 ± 1.24^a^	3–7	–	5.29 ± 1.22	3–7
Depressive symptoms (BDI)	–	7.50 ± 5.51^a^	0–26	–	7.25 ± 4.96	0–22
State anxiety symptoms (STAI-State)	–	31.55 ± 7.63^a^	20–55	–	31.49 ± 7.27	20–54
Parenting stress	–	60.05 ± 14.92^a^	38–99	–	59.65 ± 13.92	39–88
History of depression or anxiety diagnosis (Yes)	26 (33.8)	–	–	23 (39.0)	–	–
Anxiety and depression medication use (Yes)	5 (6.5)	–	–	4 (6.8)	–	–
Interval between home and fMRI visits (months)	–	–	–	–	1.02 ± 1.04	0.07–6.33
Breastfeeding	47 (61.0)			39 (66.1)		
Right handedness	–	–	–	50 (84.7)	–	–
Relationship Status (Married/Engaged/Common Law Marriage)	62 (81.6)			48 (82.8)		
Time away from own infant per week (hours)	–	14.04 ± 15.31	0–50	–	13.36 ± 15.39	0–50

^a^Data from one participant was missing.

### Whole-brain analysis of trait coping styles

**Figure 2:**
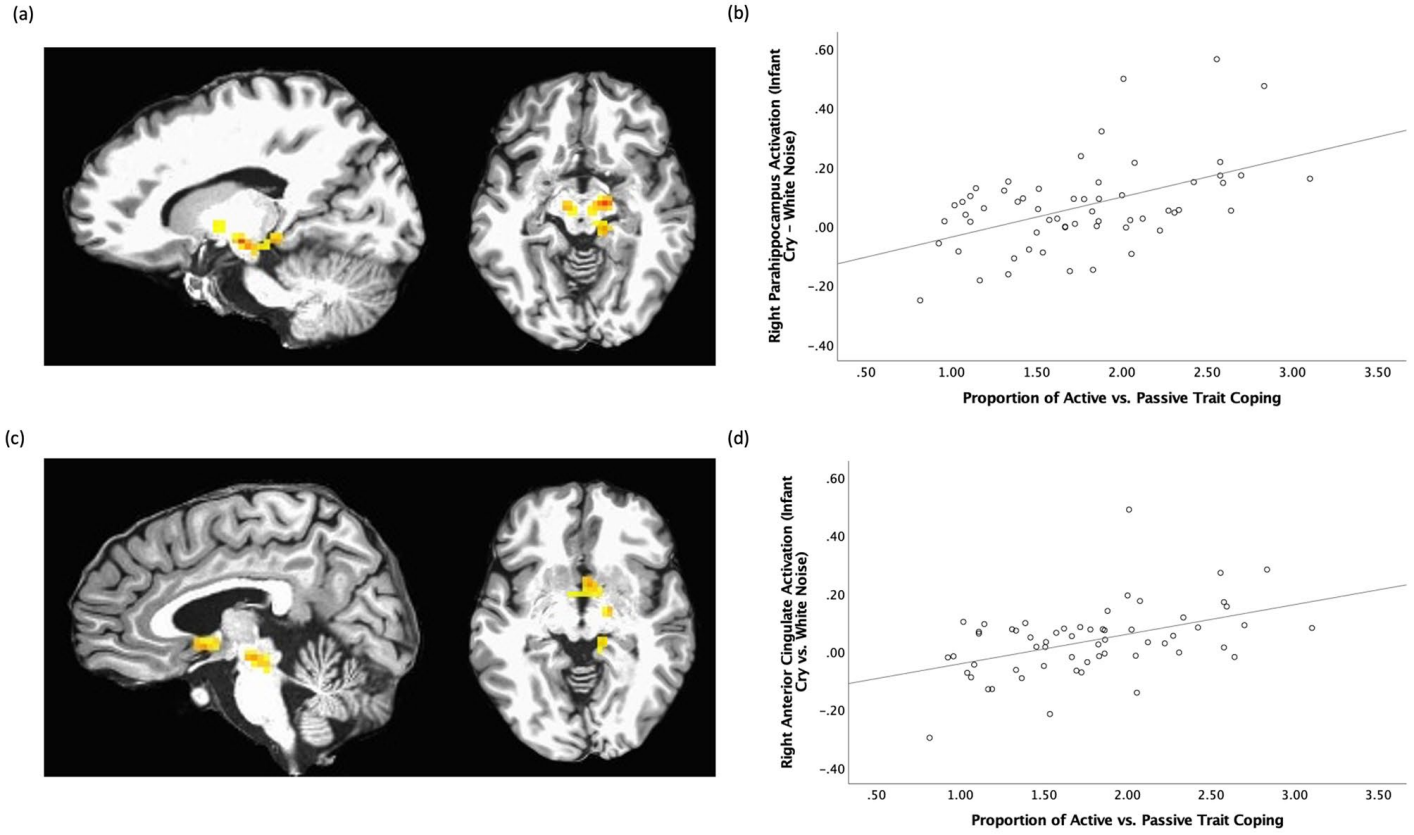
(**a**) The right parahippocampal gyrus cluster (x, y, z = 14, − 13, − 7; k = 102) which also included a part of the bilateral substantia nigra showing trait coping style X sound interaction, *p* < 0.05, corrected; (**b**) scatterplot describing the positive associations between trait coping style and brain responses to infant cry vs. white noise sounds in the region; (**c**) the right anterior cingulate gyrus (BA25; x, y, z = 5, 5, − 4; k = 38) which also included a part of the nucleus accumbens showing trait coping style X sound interaction, *p* < 0.05, corrected; (**d**) a scatterplot describing the positive associations between trait coping style and brain responses to infant cry vs. white noise sounds in the region.

In the participants with fMRI data (N = 59), the group-level whole-brain analysis revealed a two-way interaction of coping style X sound (cry, white noise). There were four clusters—(1) the right parahippocampal gyrus that included the bilateral substantia nigra, (2) left cerebellum, (3) right inferior frontal gyrus, and (4) right anterior cingulate cortex including nucleus accumbens (Table 2, Fig. 2). In all clusters, the greater proportion of active/passive coping styles was associated with greater activation in response to infant cry sounds (both own and control infant cry sounds) compared to matching white noise sounds.

**Table 2:** Brain areas showing Coping Style X Sound (cry, white noise) interactions.

Regions	BA	Side	x	y	z	Cluster size	F
Parahippocampal Gyrus	–	R	14	− 13	− 7	102	22.90
Cerebellum	–	L	− 28	− 73	− 37	67	20.17
Inferior frontal gyrus	47	R	35	23	− 19	39	20.81
Anterior cingulate gyrus	25	R	5	5	− 4	38	17.48

*p* < 0.05, corrected; BA, Brodmann area, R, right, L, left; the Talairach coordinates, and F-statistics represent the voxel with maximum signal intensity (i.e. peak value) for each cluster.

We followed up with an analysis to confirm whether the associations were driven by brain responses to infant cry or white noise sounds. In all clusters, the greater proportion of active/passive coping styles was associated with greater brain responses to infant cry (both own and control infant cry sounds), *rs*(57) > 0.35, *ps* < 0.007. The proportion of active/passive coping styles was not associated with brain responses to white noise sounds (both own infant cry matched and control infant cry matched white noise sounds), *ps* > 0.10.

### Association between brain responses to infant cry and parenting

**Figure 3:**
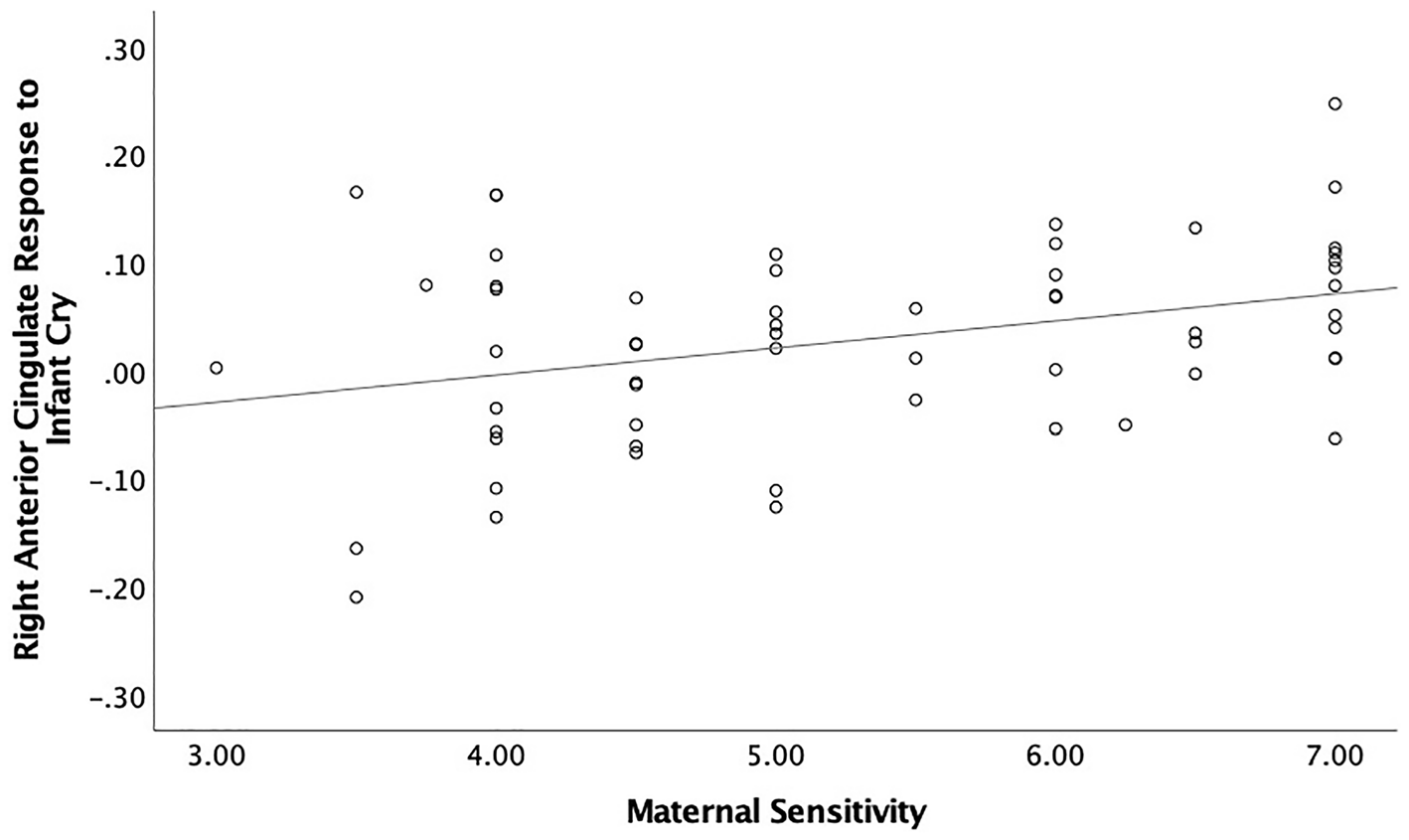
Scatterplots describing the positive associations between maternal sensitivity and brain responses to infant cry sounds (both own and control infant cry sounds) in the right anterior cingulate gyrus (BA25; x, y, z = 5, 5, − 4; k = 38; Table 2) and maternal sensitivity.

A regression analysis was performed to test the association between brain responses to infant cry sounds and maternal sensitivity that was observed during interactions with own infant. Brain response to infant cry in the right anterior cingulate cortex was positively associated with maternal sensitivity, *B* = 0.34, *p* = 0.008, *q* < 0.05 (Fig. 3). Brain responses also explained a significant proportion of variance in maternal sensitivity scores, *R*^2^ = 0.12, F(1, 57) = 7.45, *p* = 0.008. Brain responses to infant cry in other regions were not associated with maternal sensitivity, *Bs* < 0.23, *ps* > 0.09.

## Discussion

The postpartum period is often a stressful and demanding time for new mothers, who can be vulnerable to depression and anxiety as well as negative feelings about parenting^53^. Therefore, it is important to understand what resilience factors can support a mother’s psychological well-being and positive parenting behaviors. The current study examined the associations between trait coping styles and mothers’ neural and psychological adaptation to motherhood. We report that more active coping styles compared to passive coping styles are associated with adaptive psychological and neural outcomes in mothers. In the whole sample, the greater use of active vs. passive coping was associated with lower anxiety, depressive symptoms, and parenting stress. In the fMRI sample, higher proportion of active vs. passive coping styles was associated with greater brain activation in response to infant cry sounds in brain regions that are involved in maternal motivation and emotion regulation such as substantia nigra, anterior cingulate cortex (ACC), and inferior frontal gyrus (IFG). Although coping style was not directly associated with parenting behaviors, greater brain activation in these regions was associated with more sensitive parenting. Therefore, the results suggest that coping style is significantly associated with maternal brain sensitivity to infant cues, which is then further associated with sensitive maternal behaviors during the postpartum period.

In the whole sample (N = 77), consistent with the literature, we found that more active coping styles compared to passive coping styles was adaptive during the postpartum period. The mean value of the proportion of active vs. passive coping styles was 1.76 (Table 1; 1 would indicate the same proportion of active to passive coping styles), which suggests that on average mothers use active coping styles more often than passive coping styles. However, we also found that there was a relatively wide range in the ratio of active/passive coping styles, and some mothers with the coping style value below 1 were using passive coping styles more dominantly than active coping styles. Furthermore, consistent with the hypothesis and findings of previous studies^1,19^, we found that the greater proportion of active to passive coping styles was associated with lower depressive mood and anxiety symptoms as well as lower levels of parenting stress. In general, active coping styles are considered a protective factor for the negative impact of stress exposure^54^. Thus, the current study identifies greater proportional use of active vs. passive coping strategies as a protective factor for mothers during the postpartum period.

In the subset of the sample with fMRI data (N = 59), the associations between trait coping style and postpartum outcomes were weaker. Because average scores for coping style, mood, and parenting variables did not differ between the overall sample and the subset of participants included in the fMRI analysis, the most likely reason for the weaker associations was due to the smaller sample size. However, in this sample, we found that mothers’ trait coping styles were associated with their brain responses to infant cry sounds compared to white noise sounds. The greater use of active vs. passive coping was associated with increased brain responses to infant cry across own vs. control baby cry sounds. The brain regions that showed associations with the coping style included some of the key areas involved in parental motivations and behaviors. The limbic brain regions including the parahippocampus and substantia nigra, the anterior cingulate gyrus and nucleus accumbens comprise the mesocorticolimbic dopaminergic reward/maternal motivation circuit that critically supports maternal motivation^31,55^. Along with the anterior cingulate cortex, the specific region of the interior frontal gyrus that was identified in the analysis was also referred to the ventrolateral PFC (BA47) and it is involved in emotion regulation^51^. Several meta-analyses have identified these regions to be consistently activated during mothers’ responses to infant cues^56–58^. Furthermore, increased brain activation in these regions has been associated with more sensitive parenting behaviors among mothers^33,36–40^. The current findings suggest that mothers who use more active vs. passive coping styles demonstrate greater brain activations in key maternal motivation and emotion regulation regions in response to infant cues.

The findings raise questions about potential mechanisms underlying the observed associations. One possibility is that the use of active coping strategies supports more effective emotion regulation. The coping strategies assessed in the current study are trait-related coping across situations. Trait cognitive reappraisal and reframing have been associated with reduced physical and emotional reactivity to stress^59,60^. Thus, mothers who use more active coping strategies may have an enhanced ability to regulate their stress and negative emotions across stressful situations. This may have been associated with increased activation in the brain regions that are associated with emotion regulation in response to infants’ distress cues. On the other hand, in other studies mothers’ increased exposure to chronic stress or childhood trauma was associated with more dampened brain responses to infant cues in the inferior frontal gyrus and cerebellum^7^. Exposure to stress is also a risk factor for lower behavioral sensitivity in mothers^5,6,25^. Recent studies suggest that interventions focused on reducing mothers’ stress and supporting their parenting skills can lead to enhanced functional connectivity between the amygdala and subgenual anterior cingulate cortex in response to infant cry sounds^61^. These studies highlight the importance of effective emotion regulation ability for parental responses to infant cues. Trait coping strategies support emotion regulation ability and further are associated with increased activation in the neural regions that are involved in emotion regulation in response to negative infant cues.

A second possibility is that the greater use of active vs. passive coping styles leads to less negative mood. Depressive symptoms have been associated with dampened brain activation to infant cry sounds in nucleus accumbens and the inferior frontal gyrus^62^, and reduced functional connectivity between the amygdala and nucleus accumbens in response to infant cry sounds^63^. Therefore, among mothers who used more active coping styles, lower anxiety and/or depressive symptoms may support stronger brain activation to infant cues in regions that support maternal motivation and behaviors. However, in the current study, it should be noted that the associations between coping styles and brain activation was detected after controlling for mood symptoms.

A third potential mechanism may involve neural systems that simultaneously support active coping and parental motivation. Active coping styles tend to be supported by more approach-oriented behavioral responses to stressful situations and strategies to enhance positive emotions^54,60^. Thus, it has been speculated that active coping styles are supported by the mesocorticolimbic dopaminergic reward processing and motivation circuit^42,54^. Therefore, mothers who rely on active coping strategies may have a greater tendency to activate the reward processing and motivation brain circuit when they are exposed to stress. This may further support greater brain activations to infant distress cues in the circuit that includes accumbens and substantia nigra among mothers in the current study.

Approach-oriented brain responses to infant cry may further support behavioral sensitivity during interactions with one’s own infant. Maternal behavioral sensitivity was assessed based on warm and caring behaviors toward the infant including maternal affect, speech, and touch^64,65^. These are key approach behaviors that are supported by activations of the motivation brain circuits in both human mothers and non-human animals^66,67^. We should note that coping styles were not directly associated with maternal sensitivity. One possibility is that coping styles reflect reactions to stress in general, whereas brain responses specific to infant cry sounds may be a more sensitive measure of individual differences in maternal behaviors. This, however, highlights one of the limitations of the current study: coping styles were not assessed specifically in response to parenting circumstances. Future studies should consider coping styles in response to parenting-specific stressors. This may provide insight into the direct associations between specific coping styles and parenting behaviors.

It should also be noted that the brain responses associated with coping style were elevated across both own and control infant cry sounds and were therefore not specific to one’s own baby. Previous studies report that maternal brain responses to infant cry sounds are similar to maternal brain responses to images of one’s own baby and control baby stimuli^68^. In our previous studies with samples overlapping with the current sample, associations between maternal variables and brain responses to infant cry sounds were found across all infant cry sounds rather than specific to a mother’s own infant cry sounds^30,69^. The reasons for more limited differences between own vs. control cry sounds may include the high levels of urgency and distress that both own and control cry sounds convey. In other words, the maternal brain may respond to all infant cry sounds in a sensitive way^7^.

Future studies will be important to understand what contributes to individual differences in coping styles. Chronic exposure to stress has been associated with learned helplessness and lower internal locus of control^70–72^, which can lead to greater use of passive and maladaptive coping styles compared to adaptive coping styles^73,74^. Genetic contributions to coping styles have also been reported^54,75^. During the postpartum period, mothers also experience changes in their hormonal levels such as cortisol and oxytocin^26,27,66,76^. These hormones are involved in parenting behavior but also in stress and emotional regulation, therefore they are likely to be associated with mothers’ coping styles during the postpartum period. Thus, studies that focus on examining genetic, biological, and environmental factors associated with coping styles among new mothers are needed. Such an understanding will also be important for the design of interventions to support mothers in using more active coping styles.

There are limitations of the current study. First, because the study is cross-sectional, it is not possible to draw conclusions on the directionality among the different variables. For example, negative mood and anxiety during pregnancy can impact both coping style and parenting outcomes. Therefore, it will be important for future studies to examine whether maternal coping styles are prospectively associated with parenting variables. Second, the current study includes a wide range of postpartum months. Mothers report higher levels of depressive and anxious symptoms during the first few months postpartum^28^, and this may be a time when coping styles are particularly important. On the other hand, later postpartum months have been associated with greater brain structural increase and functional connectivity in new mothers^77,78^. While postpartum months did not have an association with coping styles or brain activations in the current study, it will be important to examine whether coping styles during the first few months postpartum or even during pregnancy may be particularly important to parenting. Third, although the current study included relatively ethnically diverse mothers with a range of SES backgrounds, the sample size was limited to examine the impact of culture on coping styles and parenting behaviors^79,80^. Also, most of the mothers were not clinically depressed or anxious and were relatively well-adjusted to motherhood at the time of the study. Information on the chronicity of depressive symptoms was missing; thus, the current study does not speak to whether the coping styles are associated with elevated depressive symptoms due to major depressive disorder or postpartum depression. Studies that include mothers who are at greater risk, such as those with clinical levels of postpartum depression or anxiety, will be important for developing an understanding of the role of coping styles in protecting mothers from stressful environments.

In the current study, we examined whether coping styles were associated with brain and behavioral responses to infants among new mothers. Consistent with previous literature, we found that in the whole sample of new mothers, more use of active coping styles such as positive reframing and seeking support from others was associated with less negative mood and anxiety and more sensitive parenting compared to passive coping styles such as denial and venting. The findings further provided information that can contribute to understanding neurobiological pathways by which coping styles can help mothers’ adjustment to parenthood. We found that the greater proportion of active vs. passive coping styles was associated with greater levels of brain responses to infant cry sounds compared to white noise sounds in brain regions that are important for maternal motivation and empathetic responses. Greater brain responses in the midbrain regions and ACC were further associated with more sensitive parenting behaviors. The postpartum period is a stressful time due to a multitude of challenges—changes in life priorities, demands of taking care of an infant, and other material and time constraints that parents need to navigate. The findings of the current study suggest that in addition to emotional and instrumental supports, supporting mothers in using active coping styles can help to promote resilience during the postpartum period and empower mothers to develop positive long-term relationships with their infants.

## Methods

### Participants

Seventy-seven first-time mothers with a healthy infant participated in the current study. The participants were recruited via flyers and brochures in postpartum clinics in the Denver metro area, as well as in the offices of federal and state programs that serve low-income new families (i.e. the Women, Infant, and Children (WIC) clinics, and Colorado-state’s Prenatal Plus Programs). All participants were first-time mothers, age 18–40, English speaking, and had full-term birth without major birth or pregnancy complications. They had IQ scores above 70 [based on WASI-II (Wechsler Abbreviated Scale Intelligence)], were free of a history of psychiatric diagnosis other than depression or anxiety (based on self-report), had a family income-to-needs ratio below 8, and their infants did not stay in the NICU longer than one day after birth. Inclusion of participants with a history of depression or anxiety disorders ensured that the sample in the current study would be representative of the population, as these two disorders are relatively common. Mothers with very high income were excluded to increase representation of low- and middle-income mothers in the study.

Among seventy-seven participants, 61 participated in the neuroimaging part of the study. Sixteen participated in only the home visit part of the study because they were not eligible or were not interested in the neuroimaging part. Additional criteria for the neuroimaging part of the study included no metal in the body and no known claustrophobia. Of the sixty-one participants who participated in the neuroimaging part, two were excluded from the analysis. One participant was removed due to excessive motion (see the fMRI processing), and another was removed due to a technical error during scanning (i.e. use of incorrect stimuli). An overlapping sample of fMRI data from the current study has been included in prior publications^30,69,81,82^, however, the current research question has not been examined.

A priori power analysis using G*Power Version 3.1^83^ demonstrated that a sample size of 32 would allow for the detection of effect size (f = 0.25) with 95% power at an alpha of 0.05 for the repeated measures with two within-subject factors with two conditions each and one between-subjects factor. Thus, the current sample size (N = 59) resulted in sufficient power to detect a medium to high effect size. The posthoc analysis of effect size conducted using G*Power Version 3.1 indicated that the whole sample (N = 77) correlation between coping style and mood/parenting variables had an effect size of f = 0.38 and the fMRI sample (N = 59) regressions between brain activation and maternal sensitivity had an effect size of f = 0.43. The result confirmed that the current sample size resulted in sufficient power to detect high effect size.

### Procedures

Individuals who were interested in the current study contacted the research lab. After confirming that the individuals met the eligibility criteria, the home visit was scheduled. The study protocol was approved by the Institutional Review Board of University of Denver. Informed consent was obtained from all participants prior to participation. All methods and procedures were carried out in accordance with relevant guidelines and regulations. During the home visit, at least two trained researchers visited the participant’s home. Researchers collected demographic information, interviews, and questionnaires as well as a video recording of mother-infant interactions. Samples of natural infant cry sounds were also obtained. On average within a few weeks after the home visit (Table 1), the mothers were asked to visit the neuroimaging center. During the neuroimaging part of the study, mothers received training for the fMRI tasks and participated in the neuroimaging scan and post-scan tasks. Participants were compensated with cash at the end of each visit.

### Measures

#### Trait coping styles

Coping styles were assessed using the Brief COPE (Coping Orientation to Problems Experienced Inventory)^84^. The questionnaire assesses trait coping by asking ways to cope with stress in life across situations and time^85^. The self-report questionnaire included 28 items, scored from one (“I haven’t been doing this at all”) to four (“I’ve been doing this a lot”). The measure included 14 subscales (2 items for each subscale) including active coping, planning, use of instrumental support, positive reframing, acceptance, use of emotional support, denial, venting, self-blame, humor, religion, self-distraction, substance use, and behavioral disengagement. The sum score of the two items indicated the score of each subscale.

Principal component analysis with varimax rotation was conducted^74^. Two criteria were applied: (1) items were loaded > 0.03 on one of the factors and (2) their loading was positive. If an item was loaded > 0.03 on more than one factor, the item was assigned to the factor with a higher loading value. The results suggested that the coping items were loaded into two factors—active and passive coping styles. Active coping styles included active coping, emotional support, instrumental support, reframing, planning, humor, accepting, self-distraction, and religion. Passive coping styles included denial, substance use, disengaging, venting, and self-blame. A similar two-factor approach has been used with both perinatal samples and other populations^86–94^. All strategies in the active coping group are typically considered to be adaptive, including self-distraction^95,96^. When an individual has little control over the situation, self-distraction can be a protective active coping strategy^97^. All items included in the passive coping group are typically associated with negative outcomes^95^. Cronbach’s alpha was 0.85 for the active coping group and 0.57 for the passive coping group.

The mean score of all items of each style was first calculated. Then, the ratio of active vs. passive coping was calculated to create a coping style variable. The absolute number of coping responses differed among individuals—some participants reported using more strategies than other participants. The ratio approach was used to create a more standardized variable across participants^74,98^.

#### Depressive and anxiety symptoms

Depressive symptoms were assessed using the Beck Depression Inventory (BDI)^99^. The self-reported questionnaire includes 21 items that ask about symptoms during the past week. Each item was rated based on a 4-point Likert scale, from “absent (0)” to “severe (3)”^100,101^. In the whole sample, 24% (N = 18) reported mild depressive symptoms (BDI score = 10–18) and 4% (N = 3) reported moderate depressive symptoms (BDI score = 19–29). In the fMRI sample, 29% (N = 17) reported mild depressive symptoms and 2% (N = 1) reported moderate depressive symptoms. Cronbach’s alpha was 0.80.

Anxiety symptoms were assessed using the STAI (The State-Trait Anxiety Inventory) –, State anxiety section ^102^. The self-reported questionnaire includes 20 items that ask about current anxiety-related symptoms. Each item was rated based on a 4-point Likert scale (0 = not at all, 3 = very much so). During the postpartum period, a score of 43 is the threshold for an elevated anxiety level^103^. In the current study, 11% (N = 8) of the whole sample and 10% (N = 6) of the fMRI sample reported symptoms above the threshold level. Cronbach’s alpha was 0.86.

#### Parenting variables

Three measures were included to assess adaptation to motherhood. First was the Parenting Stress Index—short form^104,105^. This self-reported questionnaire includes 36 items on a 5-point Likert scale (1 = strongly disagree, 5 = strongly agree). It consists of three subscales: Parental Distress, Parent–Child Dysfunctional Interaction, and Difficult Child. Cronbach’s alpha was 0.89.

Second, mother-infant interactions were video-recorded and coded for maternal sensitivity. Mother participants were asked to naturally interact with their infants as they would every day for 15 min. Researchers left the room after the recording had been started. Mother participants were asked not to use toys and to avoid feeding the infants. Researchers who were blind to the participant’s demographic information and who were trained and certified by Dr. Biringen, creator of the Emotional Availability scales, coded maternal sensitivity using the Emotional Availability Scales 4th edition^64,65^. The maternal sensitivity scale assesses the caregiver’s ability to provide warm and emotionally connected care based on observations of maternal affect, clarity, timing, flexibility, acceptance, amount, and conflict. The range of the scale was 1–7 with higher scores reflecting optimally sensitive maternal behaviors. Two coders coded the videos, with 24% overlap. The average intraclass correlation (ICC) was 0.91.

### fMRI measure

#### Infant cry paradigm

Maternal brain responses to infant cues were assessed using an fMRI task that included infant cry sounds. The task included two types of cry stimuli—mothers’ own baby cry sounds and a control baby cry sound that was the same across all participants. The own cry samples were recorded while the cry was naturally happening during the home visit using a digital voice recorder. The home visit lasted 3–4 h so there were opportunities to record infant cries during that period. Our records indicate that the cries largely related to being hungry (50%), seeking attention (29%), and a normal range of discomfort (e.g. diaper change or being placed on a weighing scale; 11%). Thus, no own cry samples have been recorded outside of a typical range of pain (e.g. heel stick, circumcision). The control baby sound has been rated as having an average level of emotional intensity by independent raters. The cry sound samples were edited using Cool Edit Pro Version 2.0 (Syntrillium Software, Phoenix, AZ) to remove unrelated noise and then to create a 20-s cry sound clip. The sound volume was matched between control and own baby sound stimuli. Next, the white noise stimuli for both own baby cry sounds and control cry sounds were synthesized by generating a spectral average of the cry, which was then matched to the gross temporal envelope of the own infant and control infant cry sounds.

The fMRI task thus included four types of sounds—own baby cry sound, own baby cry matched white noise sound, control baby cry sound, and control baby cry matched white noise sound. Inside the scanner, mothers listened to the sounds through noise-cancelling headphones. The sound volumes were consistent across participants. Each of the sounds was 20 s long and each was presented 10 times in a randomized order across two functional runs. During the sound block, participants were asked to let their feelings and thoughts occur naturally. There was an average 10 s rest period (ranged 8–12 s) between each sound block when a fixation cross was on the screen and no sound was presented. The task was 21 min in total duration.

#### fMRI parameters

Due to a scanner upgrade during the study, both 3.0T Siemens Trio scanner (N = 36) and 3.0 T Siemens Prisma scanner (N = 23) were used. A T2*-weighted gradient echo-planar imaging (EPI) image was acquired using matching parameters between the scanners (540 volumes; TR = 2300 ms; TE = 27 ms; flip angle = 73°; field of view = 192 mm^2^; matrix size, 64X64; 36 axial slices; voxels = 3 mm^3^). We examined the potential differences in data between the two scanners. The mean average temporal signal-to-noise ratio (TSNR) did not significantly differ by scanner type, *t*(57) = 0.35, *p* = 0.73 (Siemens Trio: *M* = 226.99, *SD* = 31.26; Siemens Prisma: *M* = 223.69, *SD* = 42.31). Thus, we did not find strong evidence for systematic scanner effect in the brain. However, to address a concern regarding data from two different scanners, we included scanner type as a covariate in the whole-brain model.

High resolution anatomical T1-weighted images using the 3D magnetization-prepared rapid gradient-echo (MPRAGE) protocol were acquired. For the Trio scanner, the parameters were 192 sagittal slices, TR = 2530 ms, TE = 1.64 ms, flip angle = 7°, FOV = 256 mm^2^ and voxels = 1 mm^3^. For the Prisma scanner, the parameters were 224 sagittal slices, TR = 2400 ms, TE = 2.07 ms, flip angle = 8°, FOV = 256 mm^2^ and voxels = 0.8 mm^3^.

#### fMRI preprocessing

Preprocessing and analysis of all image data was conducted using Analysis of Functional Neuroimages software (AFNI version 18.3.12)^106^. Preprocessing steps followed the standard procedures in afni.proc.py. The first two TRs (in addition to 4 dummy TRs) of each run were discarded to account for magnetic equilibrium. The following steps included slice timing correction, distortion correction, coregistration, warping to Talairach space, spatial smoothing with a 6-mm root-mean-square deviation Gaussian blur, and scaling into percent signal change. Individual volumes that had motion greater than 0.5 mm in any direction or more than 10% of voxels as outliers were centered. One participant who had more than 20% of the total number of volumes censored was excluded from the analysis. Among participants included in the analysis (N = 59), the range of number of TRs censored was 0–97 (M = 14.02; 2.6% of the total volumes). At the individual participant level, general linear models (GLM) using regressors of four conditions (boxcar function convolved with hemodynamic response function), third-order polynomials, and 6 motion parameters were used.

### Analysis

#### Correlations between trait coping style, maternal mood, and parenting

Associations among main and demographic variables in all mothers (N = 77) as well as the subset of mothers with fMRI data (N = 59) were analyzed. For continuous variables, the bivariate Pearson correlation or Spearman’s correlation (if the variables were nonnormally distributed) were used. For categorical variables, Chi-squared test was used. We corrected for multiple testing using the Benjamini–Hochberg procedure^107^ treating mood variable models (BDI, STAI) and parenting variable models (PSI, YIPTA, Senstivity, Non-Intrusiveness) as separate analyses. We used a False Discovery Rate (FDR) correction of *q* < 0.05.

#### fMRI analysis

At the group-level of fMRI analyses, AFNI’s 3dLME was utilized to create a whole-brain linear mixed-effects model with the proportion of active/passive coping style as a between-subjects factor, and sound (cry, noise) and condition (own, control) as within-subject factors. Postpartum months and scanner type were included as covariates as they may influence maternal brain responses to infant cues. An EPI-based mask (90% overlap across participants) was applied to exclude data outside of the brain from the analysis. AFNI's 3dClustSim program using the spatial autocorrelation function (ACF) option was used for multiple comparison corrections. The cluster extent threshold was k ≥ 33 with a height threshold of *p* < 0.001, equivalent to a whole-brain corrected false positive probability of *p* < 0.05. The activation data extracted from each cluster identified in the whole-brain analysis were imported into a SPSS dataset.

The extracted data were used in the analysis to decompose interaction findings. First, brain activation data from each condition (own infant cry, control infant cry, own infant cry matched white noise, and control infant cry matched white noise) was extracted using the masks of the functional clusters. Then, the average percent signal change values across the two cry conditions and across the two white noise conditions were included in the following correlation analyses with the proportion of active/passive coping style variable.

Regression analyses were performed to examine the associations among brain responses to infant cry and maternal sensitivity. Separate analyese for the four clusters indentified in the whole-brain analyses were conduted. We corrected for multiple testing using the Benjamini–Hochberg procedure^107^. We used a False Discovery Rate (FDR) correction of *q* < 0.05.

## Supplementary Information

Supplementary Information.

## Data Availability

The data that support the findings of this study are available from the corresponding author upon reasonable request.
