# Supplementary material for: Trait coping styles and the maternal neural and behavioral sensitivity to an infant
Source: Sci Rep. 2022 Aug 23;12:14373. doi: 10.1038/s41598-022-18339-w (PMC9399102; doi:10.1038/s41598-022-18339-w)
Supplement: Supplementary file 1 — Supplementary Information. [file 41598_2022_18339_MOESM1_ESM.docx]

**Analysis of differences between the sample included in the fMRI analysis (N=59) and not included in the fMRI analysis (N=18).**

Differences between the two groups were examined using independent t-tests and Pearson chi-square tests. Across all variables listed in Table 1, the only difference between the two groups was infant sex. The group that was not included in the fMRI analysis had proportionally more male than female infants (female/male = 5/13) compared to the group that was included in the fMRI analysis (female/male = 35/24), *X*^2^ (1, N = 77) = 5.50, *p* = 0.02.

**Supplementary Table 1**

***Correlation Table – Full Sample***

|  | 1 | 2 | 3 | 4 | 5 | 6 | 7 | 8 | 9 | 10 | 11 | 12 | 13 | 14 | 15 | 16 |
| --- | --- | --- | --- | --- | --- | --- | --- | --- | --- | --- | --- | --- | --- | --- | --- | --- |
| 1.Maternal age at home visit (years) | -- |  |  |  |  |  |  |  |  |  |  |  |  |  |  |  |
| 2. Maternal Hispanic ethnicity | -.30^**^ | -- |  |  |  |  |  |  |  |  |  |  |  |  |  |  |
| 3. Maternal education (years) | .71^**^ | -.26^*^ | -- |  |  |  |  |  |  |  |  |  |  |  |  |  |
| 4. Infant sex (female) | .06 | -.17 | .05 | -- |  |  |  |  |  |  |  |  |  |  |  |  |
| 5. Postpartum month at the time of home visit | .20 | .15 | .16 | -.19 | -- |  |  |  |  |  |  |  |  |  |  |  |
| 6. Proportion of active/passive coping styles^a^ | .14 | -.06 | .14 | -.02 | -.10 | -- |  |  |  |  |  |  |  |  |  |  |
| 7. Maternal Sensitivity | .18 | -.12 | .21 | .03 | .05 | .01 | -- |  |  |  |  |  |  |  |  |  |
| 8. Depressive symptoms (BDI) | -.01 | -.12 | .05 | .00 | -.01 | -.28^*^ | .11 | -- |  |  |  |  |  |  |  |  |
| 9. State anxiety symptoms (STAI-State) | .08 | -.07 | .01 | .04 | .23^*^ | -.32^**^ | 0.13 | .70^**^ | -- |  |  |  |  |  |  |  |
| 10. Parenting stress | .06 | .05 | -.01 | -.10 | -.03 | -.32^**^ | -.07 | .58^**^ | .61^**^ | -- |  |  |  |  |  |  |
| 11. History of depression or anxiety diagnosis (Yes) | .06 | -.06 | -.05 | -.03 | .01 | -.22 | .07 | .37^**^ | .29^*^ | .32^**^ | -- |  |  |  |  |  |
| 12. Anxiety and depression medication use (Yes) | .12 | -.12 | .00 | -.06 | .08 | -.26^*^ | -.12 | .12 | .09 | .231^*^ | .37^**^ | -- |  |  |  |  |
| 13.Breastfeeding exclusively (Yes) | .16 | -.10 | .28^*^ | .24^*^ | -.11 | .01 | .03 | .02 | -.16 | .04 | -.01 | -.10 | -- |  |  |  |
| 14. Right handedness | -.16 | -.03 | -.04 | -.01 | -.09 | -.11 | .09 | .18 | .06 | .06 | .03 | -.09 | -.01 | -- |  |  |
| 15. Relationship status (Married/Engaged/Common Law Marriage) | .26^*^ | -.08 | .20 | .08 | .25^*^ | -.11 | -.03 | -.02 | .01 | -.03 | .13 | .13 | .04 | -.28^*^ | -- |  |
| 16. Time away from own infant per week (hours) | .04 | -.06 | .03 | -.08 | .32^**^ | -.03 | -.03 | .06 | .09 | -.07 | -.02 | .15 | -.16 | .11 | -.07 | -- |

^*^*p* < .05. ^**^*p* < .01.

^a^ Spearman rank order correlation reported due to the data distribution.

**Supplementary Table 2**

***Correlation Table – fMRI Sample***

|  | 1 | 2 | 3 | 4 | 5 | 6 | 7 | 8 | 9 | 10 | 11 | 12 | 13 | 14 | 15 | 16 | 17 |
| --- | --- | --- | --- | --- | --- | --- | --- | --- | --- | --- | --- | --- | --- | --- | --- | --- | --- |
| 1.Maternal age at fMRI scan (years) | -- |  |  |  |  |  |  |  |  |  |  |  |  |  |  |  |  |
| 2. Maternal Hispanic ethnicity | -.25 | -- |  |  |  |  |  |  |  |  |  |  |  |  |  |  |  |
| 3. Maternal education (years) | .73^**^ | -.28^*^ | -- |  |  |  |  |  |  |  |  |  |  |  |  |  |  |
| 4. Infant sex (female) | .15 | -.21 | .07 | -- |  |  |  |  |  |  |  |  |  |  |  |  |  |
| 5. Postpartum month at fMRI scan | .11 | .10 | .10 | -.10 | -- |  |  |  |  |  |  |  |  |  |  |  |  |
| 6. Proportion of active/passive coping styles^a^ | .16 | -.09 | .11 | -.003 | -.16 | -- |  |  |  |  |  |  |  |  |  |  |  |
| 7. Maternal Sensitivity | .15 | -.15 | .28^*^ | .10 | -.13 | .10 | -- |  |  |  |  |  |  |  |  |  |  |
| 8. Depressive symptoms (BDI) | .03 | -.19 | .06 | .08 | .08 | -.23 | .08 | -- |  |  |  |  |  |  |  |  |  |
| 9. State anxiety symptoms (STAI-State) | .11 | -.10 | .01 | .15 | .35^**^ | -.26^*^ | .02 | .60^**^ | -- |  |  |  |  |  |  |  |  |
| 10. Parenting stress | .18 | .11 | .14 | -.09 | .09 | -.21 | -.05 | .48^**^ | .55^**^ | -- |  |  |  |  |  |  |  |
| 11. History of depression or anxiety diagnosis (Yes) | .12 | -.04 | -.01 | -.12 | .21 | -.17 | .08 | .40^**^ | .29^*^ | .27^*^ | -- |  |  |  |  |  |  |
| 12. Anxiety and depression medication use (Yes) | .23 | -.11 | .14 | -.05 | .26^*^ | -.24 | -.04 | .16 | .12 | .14 | .34^**^ | -- |  |  |  |  |  |
| 13. Interval between home and fMRI visits (months | -.13 | -.05 | -.01 | .04 | .56^**^ | -.17 | -.08 | .13 | .18 | .00 | .26 | .19 | -- |  |  |  |  |
| 14.Breastfeeding exclusively (Yes) | .19 | -.04 | .33^*^ | .17 | -.11 | .004 | .03 | .01 | -.19 | .07 | -.07 | -.08 | .05 | -- |  |  |  |
| 15. Right handedness | -.14 | -.12 | -.01 | -.02 | -.17 | -.03 | .02 | .06 | -.08 | -.01 | .03 | -.10 | -.15 | .03 | -- |  |  |
| 16. Relationship status (Married/Engaged/Common Law Marriage) | .28^*^ | -.05 | .19 | .08 | .25 | -.19 | .02 | .14 | .10 | .05 | .18 | .12 | .15 | .00 | -.25 | -- |  |
| 17. Time away from own infant per week (hours) | .02 | -.08 | .07 | -.03 | .33^*^ | -.05 | -.05 | -.05 | .05 | -.13 | -.06 | .20 | .03 | -.17 | .07 | .01 | -- |

^*^*p* < .05. ^**^*p* < .01.

^a^ Spearman rank order correlation reported due to the data distribution.
